# A New Cage-Like Particle Adjuvant Enhances Protection of Foot-and-Mouth Disease Vaccine

**DOI:** 10.3389/fvets.2020.00396

**Published:** 2020-07-31

**Authors:** Juan Bidart, Claudia Kornuta, Mariela Gammella, Victoria Gnazzo, Ivana Soria, Cecilia Langellotti, Claudia Mongini, Roxana Galarza, Luis Calvinho, Giuliana Lupi, Valeria Quattrocchi, Ivan Marcipar, Patricia Zamorano

**Affiliations:** ^1^Instituto de Virología e Innovaciones Tecnológicas-IVIT, CICVyA, INTA-CONICET, Hurlingham, Argentina; ^2^Consejo Nacional de Investigaciones Científicas y Técnicas, Buenos Aires, Argentina; ^3^Instituto Nacional de Medicina Tropical, Puerto Iguazú, Argentina; ^4^Agencia de Extensión Rural Chascomus, INTA, Chascomus, Argentina; ^5^Estación Experimental Agropecuaria Rafaela, INTA, Rafaela, Argentina; ^6^Facultad de Bioquímica y Ciencias Biológicas – Universidad Nacional del Litoral, Santa Fe, Argentina; ^7^Universidad del Salvador, Buenos Aires, Argentina

**Keywords:** FMDV, ISPA, vaccine, adjuvant, protection, immune responce

## Abstract

Foot-and-Mouth Disease (FMD) is an acute viral disease that causes important economy losses. Vaccines with new low-cost adjuvants that stimulate protective immune responses are needed and can be assayed in a mouse model to predict their effectiveness in cattle. Immunostimulant Particle Adjuvant (ISPA), also known as cage-like particle adjuvant, consisting of lipid boxes of dipalmitoyl-phosphatidylcholine, cholesterol, sterylamine, alpha-tocopherol, and QuilA saponin, was shown to enhance protection of a recombinant vaccine against *Trypanosoma cruzi* in a mouse model. Thus, in the present work, we studied the effects on the magnitude and type of immunity elicited in mice and cattle in response to a vaccine based on inactivated FMD virus (iFMDV) formulated with ISPA. It was demonstrated that iFMDV–ISPA induced protection in mice against challenge and elicited a specific antibody response in sera, characterized by a balanced Th1/Th2 profile. In cattle, the antibody titers reached corresponded to an expected percentage of protection (EPP) higher than 80%. EPP calculates the probability that livestock would be protected against a 10,000 bovine infectious doses challenge after vaccination. Moreover, in comparison with the non-adjuvanted iFMDV vaccine, iFMDV–ISPA elicited an increased specific T-cell response against the virus, including higher interferon gamma (IFNγ)+/CD8+ lymphocyte production in cattle. In this work, we report for first time that an inactivated FMDV serotype A vaccine adjuvanted with ISPA is capable of inducing protection against challenge in a murine model and of improving the specific immune responses against the virus in cattle.

## Introduction

Foot-and-Mouth Disease (FMD) is an acute, highly contagious viral vesicle disease, which infects cloven-hoofed animals including livestock—cattle, pigs, sheep, goats, and buffaloes—as well as wild species—deer, antelopes, wild pigs, elephants, giraffes, and camelids (1).

The economic losses produced by Foot-and-Mouth Disease Virus (FMDV) infection in bovines and pigs are due to physical and productive deterioration rather than mortality. Indeed, mortality rates are low in adult animals, although they are often high in young ones due to myocarditis. However, for countries that export animals and their products, the most relevant economic impact is connected with restrictions on international trade (1). Routine vaccination with inactivated FMDV (iFMDV) can significantly reduce the economic impact of this disease.

FMDV has seven serotypes, known as A, C, O, Asia, SAT 1, SAT 2, and SAT 3. Different strains are used in different countries for vaccine formulation. Serotype A/Argentina/2001 (A2001), isolated in an outbreak of FMD in Argentina in 2000, was used in the present study as proof of concept (2).

In previous work, we developed an experimental murine model using FMDV O1 Campos that proved useful to evaluate the potency of FMDV vaccines. Although mice are not naturally infected by FMDV, experimental infections can be performed by intraperitoneal (ip) inoculation. In the murine model, the humoral and protective responses against FMDV in mice are correlated with cattle (3–6).

Commercial vaccines contain inactivated virus and adjuvants to boost the immune response. Adjuvants improve the immune response elicited against inactivated antigens, direct the immune response to a particular profile, increase the number of responding individuals, reduce the amount of vaccine doses, and/or allow attainment of homogeneous immune responses (7). It is of great importance to find new adjuvants that allow reducing the amount of virus in vaccines and that induce Th1/Th2 responses. Other desirable characteristics include stability and low cost. Immune stimulating complexes (ISCOMs) are capable of developing a Th1/Th2 balanced immune response, in addition to increasing cytotoxic responses (8–11). ISCOMs are spherical particles of ~40 nm in diameter, composed of phospholipids, cholesterol, and saponin, which can retain the antigen through hydrophobic interactions (8, 12). They have been applied to the development of several registered vaccines for veterinary applications (10). Recently, an empty cage-like particle formulation similar to one of this type of adjuvant, ISCOMATRIX®, was described. It was named Immunostimulating Particle Adjuvant (ISPA) and contains dipalmitoyl-phosphatidylcholine (DPPC), cholesterol (CHO), stearylamine (STEA), alpha-tocopherol (TOCO), and Quil A saponin (11, 13). This adjuvant was shown to surpass conventional adjuvants by improving humoral and cellular CD4/CD8 responses (11). Notably, it was demonstrated that vaccination with the transialidase protein of *Trypanosoma cruzi* (mTS) formulated with ISPA induced increased humoral and cellular immune responses that protected mice against challenge with these parasites (11, 13). Importantly, ISPA preparation can be easily scaled up.

In this work, we report the effect of ISPA as adjuvant for an inactivated FMDV vaccine both in a murine model and in cattle.

## Materials and Methods

### Animals

All experiments involving the use of animals were carried out according to National Agricultural Technology Institute (INTA) Ethics Manual “Guide for the Use and Care of Experimental Animals,” under protocol number 24/2016.

Male BALB/c mice, 8–12 weeks old from La Plata University, Argentina, were used.

Calves seronegative for FMDV by enzyme-linked immunosorbent assay (ELISA), ~8–10 months old, were used in the experiment.

### Virus

Binary ethylenimine (BEI)-iFMDV A/Argentina/2001 serotype (provided by Biogenesis Bago, Buenos Aires, Argentina) was used in ELISA assays and in the experimental vaccine formulation. Infectious A/Argentina/2001 serotype, provided by Argentine National Service of Animal Health (SENASA), was used for viral challenge. All experiments involving infectious virus were performed in the BSL-4 OIE (World Organization for Animal Health) facilities at the Institute of Virology, INTA.

### Infective Dose of FMDV for Viral Challenge

To select the infective dose of FMDV, serotype A, groups of 4 mice each were intraperitoneally (ip) inoculated with 500 μL of 10^1.5^ TCID_50_/mL, 10^2.5^ TCID_50_/mL, or 10^3.5^ TCID_50_/mL and monitored for viremia at 24, 48, and 72 h postinfection (hpi) as described in Quattrocchi et al. (5). Briefly, heparinized blood withdrawn at different hpi was spread onto BHK-21 cell monolayers grown in 48-well plates and incubated at 37°C in a 5% CO_2_ atmosphere. Then, cell monolayers were washed twice with sterile phosphate-buffered saline (PBS). Fresh D-MEM supplemented with 2% fetal calf serum (FCS) was added and the cells were incubated for 48 h at 37°C in 5% CO_2_. It was considered that animals were infected if the cell monolayer presented cytopathic effects after a blind passage. Clinical signs, including apathy, ruffled fur, respiratory distress, watery eye discharge, and loss of weight, were daily monitored from 0 to 96 hpi. An infective dose of 10^2.5^ TCID_50_ was selected out of the results of these experiments.

### Inactivated FMDV Dose to Vaccine Formulation

To select the iFMDV vaccine dose, dilutions of inactivated FMDV in PBS containing 1, 0.5, 0.3, or 0.1 μg in a final volume of 0.2 mL were prepared. Groups of mice (*n* = 8) were subcutaneously (sc) inoculated with these formulations and challenged with an ip injection of 10^2.5^ TCID_50_/mL of infectious FMDV, A2001 serotype, after 21 days postvaccination (dpv). Twenty-four hours later, viremia was evaluated as described earlier. Animals were considered protected if viremia was absent at this time point, as established in previous studies (4–6, 14–16). Percentages of protection were calculated as 100× (protected/challenged mice). A dose of 0.3 μg of iFMDV was selected from the results obtained because induction of 50% of protection and the adjuvant effect can be detected.

### ISPA Production

ISPA adjuvant is composed of alpha-tocopherol (TOCOP), phosphatidylcholine (DPPC), sterylamine (STEA), cholesterol (CHOL), and QuilA saponin. The ISPA particles have a cage-like structure of 73.0 ± 1.5 nm size as assessed by dynamic light scattering. First, liposomes were prepared with the final proportions of TOCOP: 0.00074% (0.017 mM), DPPC: 0.320% (4.35 mM), STEA: 0.0216% (0.8 mM), and CHOL: 0.143% (3.70 mM). The suspension was then extruded through a 50-nm-pore membrane and a QuilA saponin solution in acetate buffer was added to liposomes (6.5 mg/300 μL per mL of liposomes) and extruded through a 50-nm-pore membrane (11, 13).

### Vaccine Formulations and Vaccination Experiments

The vaccines to be applied in mice were formulated with (1) 0.3 μg of iFMDV in PBS (iFMDV) or (2) 0.3 μg of iFMDV in PBS mixed with 6 μL of ISPA (iFMDV–ISPA), in a final volume of 0.2 mL/dose. BALB/c mice were immunized with (1) iFMDV (*n* = 5), (2) iFMDV–ISPA (*n* = 5), (3) commercial vaccine (*n* = 5), (4) 6 μL of ISPA (*n* = 2), or (5) PBS (*n* = 2) by the sc route. Mice were challenged at 21 dpv as described earlier.

The vaccines used in cattle were formulated with (1) 12 μg of iFMDV in PBS, according to Mattion et al. (2), or (2) the same formulation with 1 mL of ISPA, in a final volume of 2 mL/dose. Cattle (*n* = 4, per group) were vaccinated sc at days 0 and 48 as follows: (1) iFMDV, (2) ISPA–iFMDV, or (3) commercial vaccine. The commercial vaccine consisted of a water-in-oil single emulsion containing O1/Campos, A24/Cruzeiro, A/Arg/2000, and A/Arg/2001 iFMDV and was provided by Biogénesis Bagó.

### Measurement of Total IgG and Isotypes Against FMDV by Sandwich ELISA

Total antibodies (Ab) against FMDV were assessed by ELISA as described previously (3–5) Briefly, Greiner Microlon® plates were coated ON at 4°C with anti-FMDV rabbit serum in carbonate–bicarbonate buffer, pH 9.6. After three washing steps, plates were blocked for 30 min at 37°C with polyvinylpyrrolidone blocking solution in the case of mouse sera (0.5 M NaCl/0.01 M phosphate buffer/0.05% Tween-20/1 mM EDTA/1% polyvinylpyrrolidone 30–40 K, pH 7.2) or with PBS/10% FCS in the case of bovines sera. An optimal dilution of inactivated FMDV was added in blocking solution. Plates were incubated at 37°C for 30 min. Then, serially diluted mouse sera (1:4) or bovine sera (1:5) in blocking solution were added. After 1 h 20 min incubation at room temperature, plates were washed and an optimal dilution of horseradish peroxidase (HRP)-conjugated anti-mouse IgG (H+L) (KPL®), HRP-conjugated anti-mouse isotypes (Southern Biotech, Birmingham, AL, USA), HRP-labeled goat anti-bovine IgG antibody (KPL®), or HRP-labeled goat anti-bovine IgG1 or IgG2 antibody (KPL®) was added. Plates were incubated for 1 h at room temperature and then washed. Ortho-phenylene-diamine (1,2-benzenediamine) dihydrochloride (Sigma Aldrich, St. Louis, MO, USA) (OPD)/H_2_O_2_ was used as the peroxidase substrate. Reactions were stopped by use of 1.25 M H_2_SO_4_ and A_492_ was measured in an absorbance microplate reader. Positive and negative control sera were included in every plate. The cut-off was established as the mean of the values of negative sera (*n* = 10) plus two standard deviations.

### Measurement of Total FMDV-Specific Antibodies by Liquid-Phase ELISA

A liquid-phase ELISA test was used according to Hamblin et al. (17), with modifications (1). Briefly, Greiner Microlon® plates were coated overnight at 4°C with rabbit anti-FMDV serum diluted to the optimal concentration in carbonate–bicarbonate buffer, pH 9.6. After washing with 0.05% Tween-20/phosphate buffered saline (PBST), plates were blocked with PBST/1% ovalbumin (blocking buffer) for 30 min at 37°C. Mice or bovine sera were serially diluted (1:10) in blocking buffer in separate tubes and a fixed amount of inactivated FMDV was added. After 1 h of incubation at 37°C with shaking, the virus–antibody mixtures were transferred to the blocked plates, and incubated for 1 h at 37°C. An optimal dilution of guinea pig anti-FMDV serum in PBS/2% normal bovine serum/2% normal rabbit serum was added for detection, followed by 1 h of incubation at 37°C. Plates were washed and peroxidase-conjugated anti-guinea pig IgG (Jackson ImmunoResearch, West Grove, PA, USA) serum diluted in the same buffer was added, followed by 1 h of incubation at 37°C. OPD/H_2_O_2_ was used as peroxidase substrate as described earlier and A_492_ was measured in a microplate reader. Strong positive, weak positive, and negative bovine reference sera were included in each test for validation. Antibody titers were expressed as the negative logarithm of the highest dilution of serum that causes an inhibition of color development higher than 50% in the average values of the control samples.

### Neutralizing Antibody Titers

Sera samples were examined for anti-FMDV neutralizing antibodies as described before (16). Briefly, serial dilutions of complement inactivated sera were incubated for 1 h at 37°C with 100 TCID_50_ of infective FMDV. Then virus–serum mixtures were seeded on BHK-21 monolayers. After 40 min at 37°C, fresh DMEM/2% FCS was added to the monolayers, which were incubated at 37°C, under 5% CO_2_. Cytopathic effects were observed after 48 h.

### Lymphoproliferation Assay

Murine splenocytes were obtained 21 days after immunization. Animals were anesthetized and euthanized by cervical dislocation and spleens were removed.

Cattle Peripheral Blood Mononuclear Cells (PBMCs) were obtained as described previously (18) by centrifugation of bovine blood in a Ficoll-Paque™ plus gradient (GE Healthcare, Chicago, IL, USA).

Murine splenocytes or PBMCs were labeled with 3 μM carboxyfluorescein diacetate succinimidyl ester (CFSE) in PBS for 30 min at 37°C. Labeled cells were added to 96-well plates (5 × 10^5^ cell/well) in complete RPMI 1640 media supplemented with 10% FCS and 50 mM 2-mercaptoethanol and were stimulated with (1) mock, (2) 2.5 μg/mL of iFMDV, or (3) 5 μg/mL of concanavalin A (Sigma Aldrich) as positive control. Cells were incubated at 37°C in 5% CO_2_ atmosphere for 4 days, and then 0.2% paraformaldehyde was added and cell proliferation was analyzed by flow cytometry using FACSCalibur® (Becton Dickinson, San Jose, CA, USA) and Flowing Software (Turku Center for Biotechnology, Finland). Results were expressed as delta proliferation and were calculated as the difference between the percentage of proliferating cells stimulated with inactivated virus and the percentage of proliferating cells without stimuli. An example of flow cytometry gating strategy adopted in this article is depicted in Supplementary Figure 1.

### Surface and Intracytoplasmatic Staining for IFN-γ-Producing Cells Detection

PBMC were incubated in complete RPMI 1640 media supplemented with 10% FCS and 50 mM 2-mercaptoethanol and were stimulated with (1) mock, (2) 2.5 μg/mL of iFMDV, or (3) 5 μg/mL of concanavalin A (Sigma Aldrich, St. Louis, MO, USA) as positive control. Cells were incubated for 18 h in the presence of brefeldin A (BD GolgiPlug™) (according to manufacturer recommendations). After washing, cells were fixed in 0.5% paraformaldehyde and permeated with saponin (0.1% in PBS). Permeated cells were incubated for 20 min at RT with Alexa Flour 647 anti-bovine interferon gamma (INF-γ; clone CC302, AbD Serotec, Oxford, UK) or isotype-matched control antibody. After 20 min, cells were washed twice and stained for 30 min at 4°C with anti-bovine CD4, clone CC8 (AbD Serotec) plus FITC anti-bovine IgG (polyclonal, Jackson ImmunoResearch); PE anti bovine CD 8 (clone CC63, Bio-Rad) or FITC anti-bovine WC1 (clone CC15, AbD Serotec). Cells were then washed and fixed with 0.2% paraformaldehyde. Flow cytometry was performed in a BD FacsCalibur and analyzed with Flowing Software (Turku Center for Biotechnology, Finland). An example of flow cytometry gating strategy adopted in this article is depicted in Supplementary Figure 2.

### Statistical Analysis

The GraphPad InStat® program (GraphPad, San Diego, USA) was used. Differences between groups were analyzed by applying the non-parametric Kruskal–Wallis test, followed by Mann–Whitney *U*-test for comparisons between two groups. A *p* < 0.05 was considered as an indicator of significant differences.

## Results

### Selection of the Infective Dose for Viral Challenge in Mice

**Figure 1:**
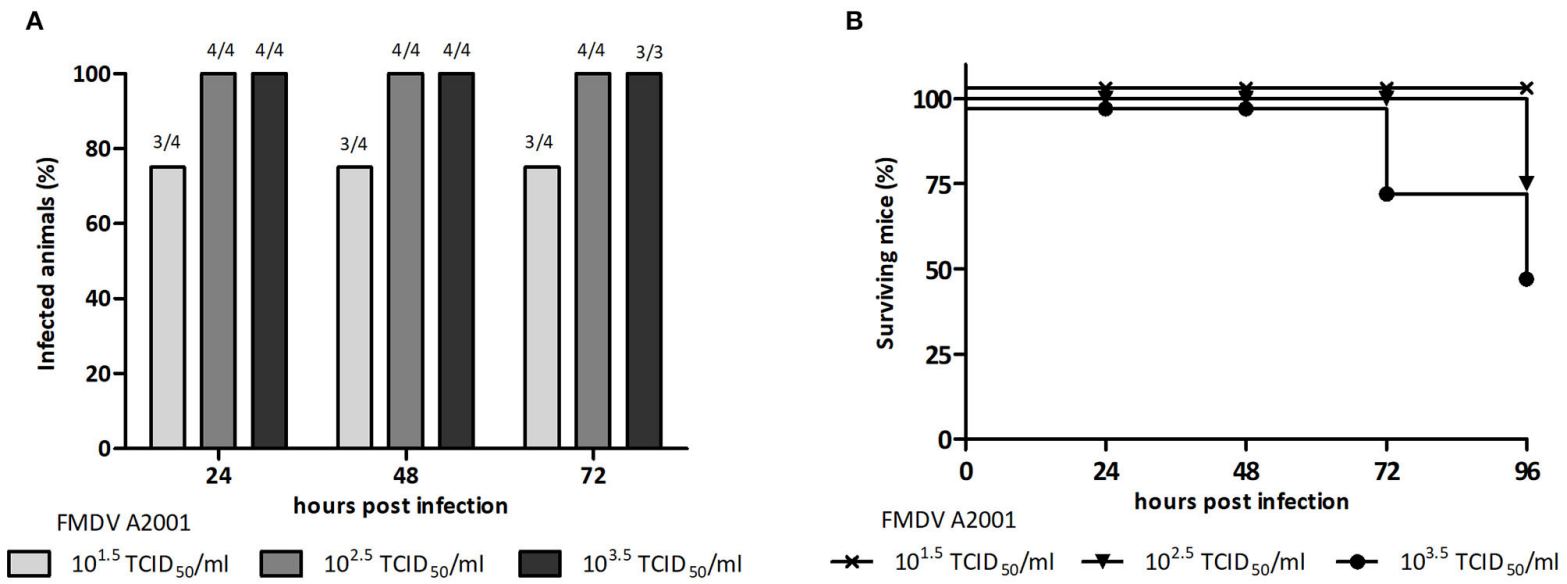
Selection of the infective dose of FMDV, serotype A. Groups of BALB/c mice (*n* = 4) were ip inoculated with 500 μL of 10^1.5^TCID_50_, 10^2.5^TCID_50_, or 10^3.5^TCID_50_/mL of infectious FMDV A/Argentina/2001, and viremia was analyzed at 24, 48, and 72 hpi. **(A)** Percentage of infected animals at 24, 48, and 72 hpi and **(B)** percentage of surviving mice at 24, 48, 72, and 96 h after inoculation with infective FMDV.

A previously developed murine model for FMDV serotype O vaccine testing was adjusted in this study to serotype A (3–5, 19). With the aim of selecting the viral challenge dose, unvaccinated mice were inoculated with different viral infective doses of FMDV (10^1.5^, 10^2.5^, or 10^3.5^ TCID_50_ infectious FMDV/mL) and viremia was assessed at 24, 48, and 72 hpi. Mice were also examined for clinical signs until 96 hpi. All mice inoculated with 10^2.5^ or 10^3.5^ TCID_50_ infectious FMDV/mL, but only 80% of those inoculated with 10^1.5^ TCID_50_ infectious FMDV/mL, presented positive viremia at all studied time points (Figure 1A). Survival was 100% at 24 and 48 hpi with all doses used. At 72 hpi, one mouse of the group inoculated with 10^3.5^ TCID_50_/mL died, and at 96 hpi, one mouse each from the 10^2.5^ TICD_50_/mL and the 10^3.5^ TICD_50_/mL groups died (Figure 1B). As shown in Table 1, clinical signs started to appear at 24 hpi in mice inoculated with 10^2.5^ TCID_50_/mL and 10^3.5^ TCID_50_/mL and at 48 hpi, all animals in these groups showed signs, including apathy, ruffled fur, and others. Conversely, no mice infected with 10^1.5^ TCID_50_/mL showed observable clinical signs at any time of the experiment.

**Table 1:** Clinical signs in mice inoculated with different FMDV A/Argentina/2001 serotype doses.

	**24 h**			**48 h**			**72 h**			**96 h**		
	**10^**1.5**^**	**10^**2.5**^**	**10^**3.5**^**	**10^**1.5**^**	**10^**2.5**^**	**10^**3.5**^**	**10^**1.5**^**	**10^**2.5**^**	**10^**3.5**^**	**10^**1.5**^**	**10^**2.5**^**	**10^**3.5**^**
Apathy	0/4	0/4	0/4	0/4	4/4	4/4	0/4	4/4	3/3^a^	0/4	3/3^a^	2/2^b^
Ruffled fur	0/4	1/4	3/4	0/4	4/4	4/4	0/4	4/4	3/3^a^	0/4	3/3^a^	2/2^b^
Respiratory distress	0/4	0/4	1/4	0/4	4/4	4/4	0/4	4/4	3/3^a^	0/4	2/3^a^	2/2^b^
Watery eye discharge	0/4	0/4	0/4	0/4	0/4	2/4	0/4	1/4	2/3^a^	0/4	1/3^a^	2/2^b^
Loss of weight	0/4	0/4	0/4	0/4	0/4	0/4	0/4	1/4	1/3^a^	0/4	2/3^a^	2/2^b^

*Results are expressed as number of mice with signs/total number of infected mice. Groups of BALB/c mice (n = 4) were ip inoculated with 10^1.5^, 10^2.5^, or 10^3.5^ TCID_50_/mL of infectious FMDV, A/Argentina/2001 serotype, and observed for disease indications at 24, 48, 72, and 96 hpi*.

a *One animal in this group died*.

b *Two animals in this group died*.

Taking into account these results, the dose of 10^2.5^ TICD_50_/mL infectious FMDV serotype A and the time point of 24 hpi were chosen to, respectively, perform and assess viral challenge assays.

### Selection of the iFMDV Dose for Vaccine Formulation With ISPA as Adjuvant

**Figure 2:**
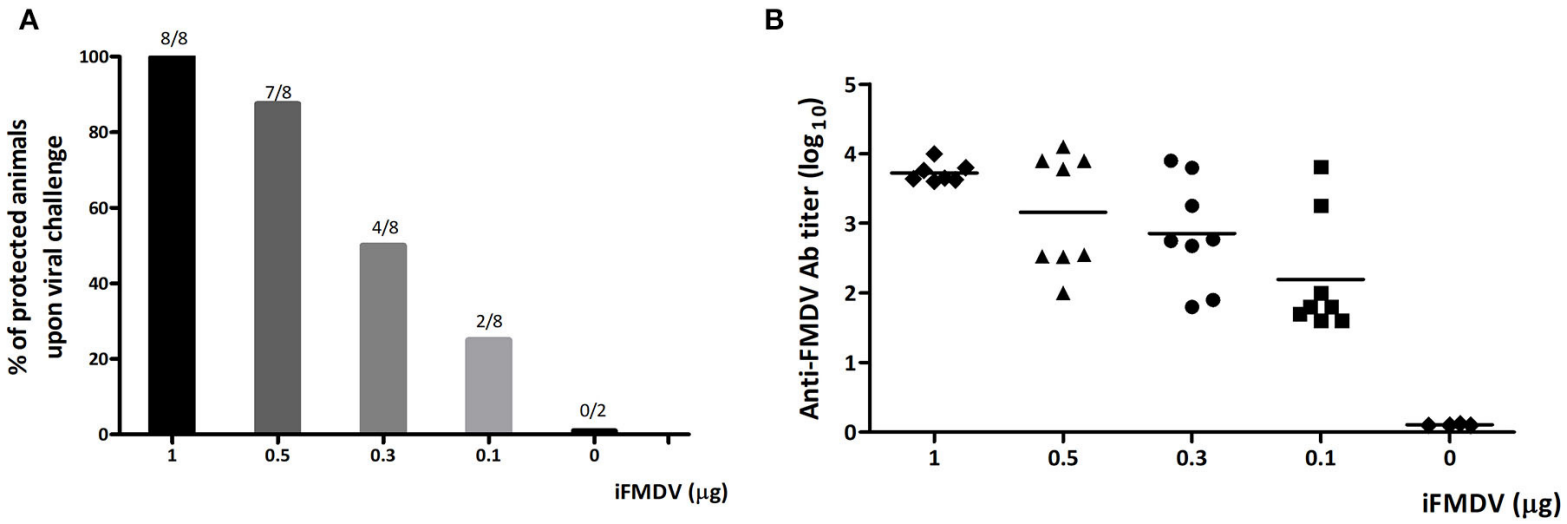
Selection of iFMDV dose for vaccination of BALB/c mice. Groups of mice (*n* = 8) were vaccinated with 1, 0.5, 0.3, or 0.1 μg of iFMDV in PBS and challenged with infective virus after 21 dpv. **(A)** Percentages of protected animals on viral challenge. Group 0 corresponds to animals inoculated with PBS. Animals were considered protected if viremia was absent at 24 h post challenge. Protection percentages were calculated as 100× (number of vaccinated animals without viremia/number of vaccinated animals). **(B)** Antibodies against FMDV elicited by vaccination with different amounts of iFMDV measured by ELISA at 21 dpv.

To analyze the modulatory effect of ISPA adjuvant on the immune response, a dose of inactivated FMDV capable of inducing 50% protection was first selected. To this end, mice were vaccinated with 1, 0.5, 0.3, or 0.1 μg of iFMDV in PBS and at 21 dpv challenged with infectious FMDV, serotype A. A dose-dependent protective effect was observed (Figure 2A), as well as a concomitant decrease in antibody titers with decreasing amounts of virus (Figure 2B). Fifty percent of mice vaccinated with 0.3 μg of iFMDV were protected upon viral challenged, so this dose was chosen for vaccine formulations.

### iFMDV–ISPA Vaccine Confers Total Protection Against FMDV in Mice With a Single-Dose Immunization

**Figure 3:**
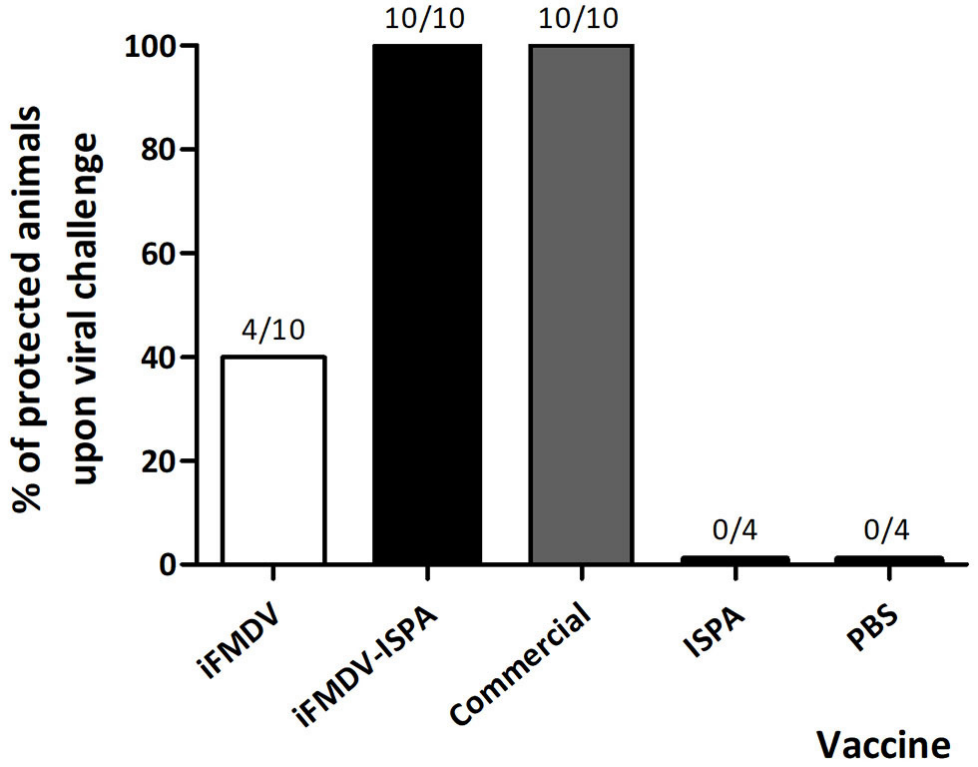
Protection on viral challenge elicited by different vaccines. Groups of mice (*n* = 10) were vaccinated with iFMDV, ISPA-iFMDV, or a commercial FMD vaccine, and groups of mice (*n* = 4) were vaccinated with ISPA or PBS alone, and challenged with infective FMDV at 21 dpv. Protection was calculated as described for Figure 2. Results are representative of two independent experiments.

The protective efficacy of the inclusion of ISPA as adjuvant in an iFMDV vaccine (iFMDV–ISPA) was tested in mice. Groups of mice were vaccinated with iFMDV, iFMDV–ISPA, a commercial vaccine (Biogénesis Bagó), ISPA, or PBS (negative control) and challenged with infective FMDV at 21 dpv (Figure 3). Notably, while protection with iFMDV alone was achieved in 40% of mice, inclusion of ISPA in the formulation increased protection levels to 100% as well as the commercial vaccine. Animals in mock vaccinated groups inoculated with ISPA or PBS were not protected, indicating that the viral challenge was conducted properly.

### Murine-Specific FMDV Antibodies and Neutralizing Antibodies Are Increased When ISPA Is Used as Adjuvant

**Figure 4:**
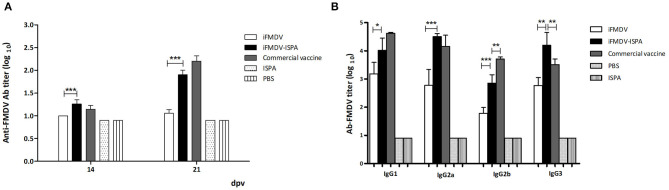
Antibodies against FMDV elicited by different vaccines in mice. FMDV specific antibody titers were measured by **(A)** liquid-phase ELISA at 14 and 21 dpv. Each bar represents the mean (*n* = 5) Ab titer ± SD in each group. **(B)** Isotype profile of vaccinated animals at 21 dpv. Data are expressed as the mean Ab titer ± SD. ***p < 0.001; **p < 0.01; *p < 0.05.

Antibody (Ab) responses elicited by iFMDV, iFMDV–ISPA, the commercial vaccine, ISPA, and PBS were evaluated at 14 and 21 dpv. Total specific FMDV Abs titers were significantly higher (*p* < 0.001) as measured by liquid-phase ELISA in the iFMDV–ISPA group as compared to the iFMDV group (Figure 4A). Importantly, when the virus neutralization test (VNT) was applied, neutralizing antibody titers at 21 dpv were significantly higher in the iFMDV–ISPA group as compared to the iFMDV group (1.6 ± 0.1 vs. 0.95 ± 0.05, *p* < 0.001). Neutralizing Ab titers in the iFMDV–ISPA group were similar to those in the commercial vaccine group (Table 2). Ab levels in the iFMDV–ISPA group were similar to those in the commercial vaccine group.

**Table 2:** Virus neutralizing antibody titers (VNT) in mice at 21 dpv with different vaccines.

**Vaccine**	**VNT (mean Ab titers ± standard deviation)**
iFMDV	0.95 ± 0.05
iFMDV–ISPA	1.7 ± 0.1^***^
Commercial	1.8 ± 0.1^***^
ISPA	<1.0
PBS	<1.0

*Titers are expressed as log_10_ of the reciprocal of the serum dilution that neutralizes 50% of 100 TCID_50_ of infective FMDV, using the fixed virus–variable serum method*.

*** *Significant differences with respect to the iFMDV group (p < 0.001)*.

Analysis of isotype profiles at 21 dpv showed that the iFMDV–ISPA group achieved higher IgG1 and IgG2a titers (*p* < 0.05 and *p* < 0.001, respectively) than the iFMDV group, and the profile was similar to that of the commercial vaccine group (Figure 4B). IgG2b titers were also higher in the iFMDV–ISPA group than in the iFMDV group (*p* < 0.001). Finally, there were significantly higher IgG3 titers (*p* < 0.001) in the iFMDV–ISPA group than in the iFMDV and the commercial vaccine groups.

### Immunization With iFMDV–ISPA Induces a Specific Cellular Immune Response Against FMDV in Mice

**Figure 5:**
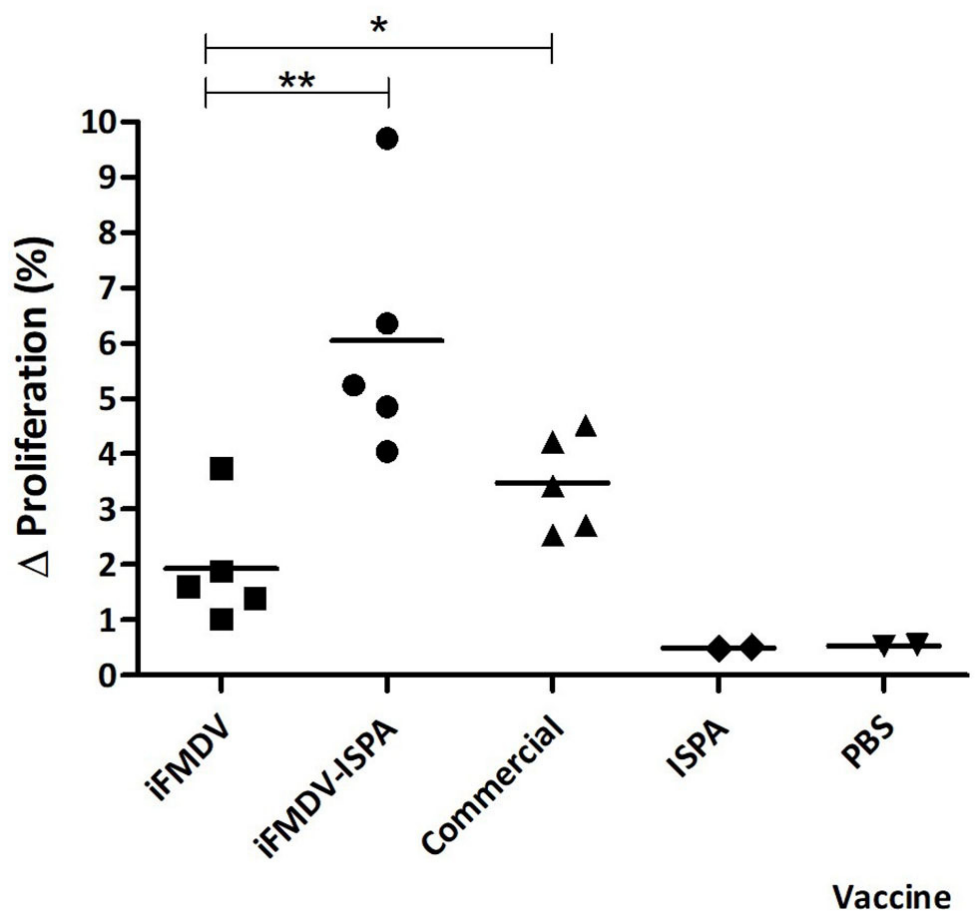
Cellular immune response in mice splenocytes at 21 dpv. Animals were vaccinated with iFMDV, ISPA-iFMDV, ISPA, or PBS. Splenocyte proliferative response after stimulation with iFMDV was measured by CFSE loss. Results are expressed as the difference (Δ%) between the percentage of proliferating splenocytes stimulated with inactivated virus and the percentage of proliferating splenocytes without stimuli. **p < 0.01; *p < 0.05.

At 21 dpv, FMDV-specific T-cell stimulation levels were significantly higher in splenocytes derived from mice immunized with iFMDV–ISPA (*p* < 0.01) or with commercial vaccine (*p* < 0.05) than in those derived from iFMDV, ISPA, or PBS-inoculated mice (Figure 5).

### iFMDV–ISPA Vaccine Induces an Increase of FMDV Abs in Cattle

After promising results obtained in the murine model, the immune efficacy of the iFMDV–ISPA vaccine was studied in cattle, a natural host of the virus.

FMDV serologically negative calves (*n* = 4 per group) were inoculated (at days 0 and 48) with iFMDV (12 μg) or iFMDV (12 μg)-ISPA, a commercial vaccine (at day 0) or PBS (negative control).

**Figure 6:**
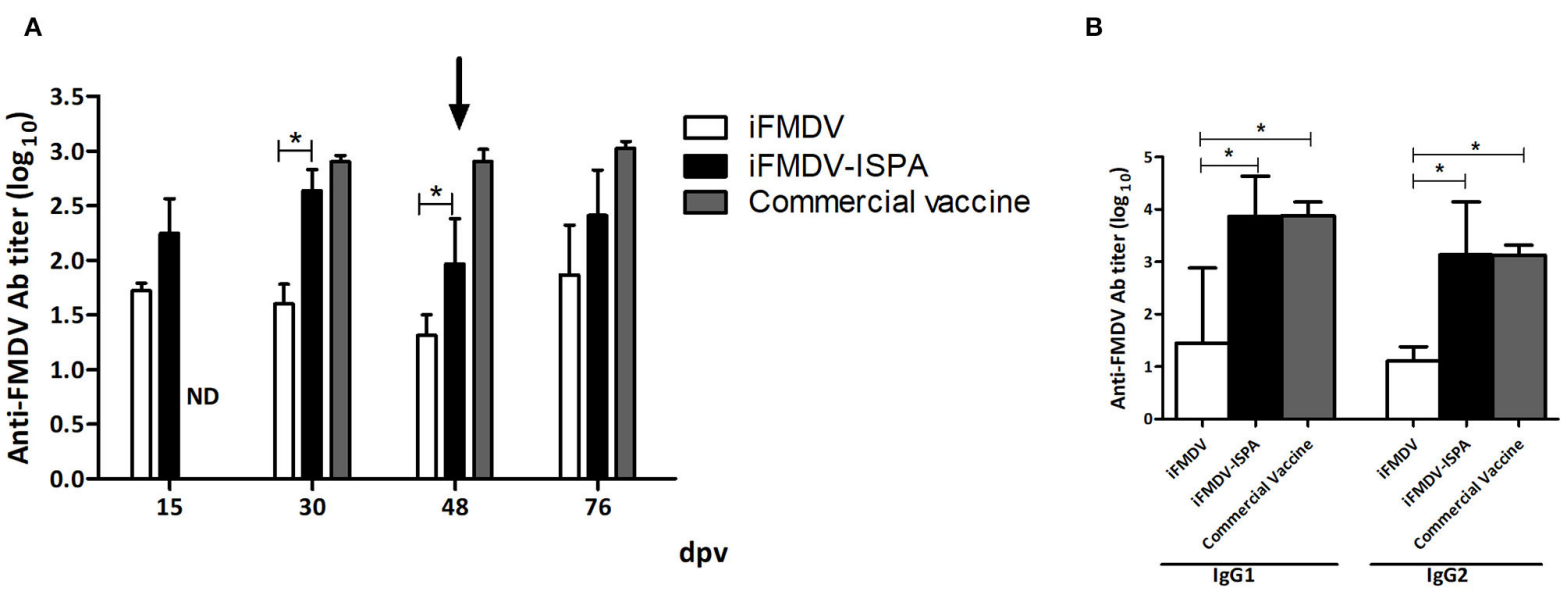
Humoral response elicited in cattle by different vaccines. FMDV-specific antibody titers were measured by liquid-phase ELISA. **(A)** Each bar represents the mean Ab titer ± SEM (*n* = 4) at 15, 30, 48, and 76 dpv. **(B)** Isotype profiles at 30 dpv, expressed as mean Ab titers ± SD. *p < 0.05.

At 30 dpv, calves vaccinated with iFMDV–ISPA displayed an increment in the elicited specific humoral response as compared to individuals vaccinated with iFMDV alone (*p* < 0.05), when measured by liquid-phase ELISA (Figure 6A).

As shown in Figure 6B, at 30 dpv, the iFMDV–ISPA vaccine induced significantly higher levels of IgG1 isotype antibodies against FMDV than the iFMDV vaccine (*p* < 0.05). Moreover, IgG2 titers also presented significant differences (*p* < 0.05) among groups. There were no statistically significant differences in isotype profiles in the iFMDV–ISPA and the commercial vaccine group.

VNT results at 30 dpv also showed a significant increase (*p* < 0.05) in Ab titers in the iFMDV–ISPA group as compared to the iFMDV group (Table 3). However, at 48 dpv, decreases in total and neutralizing Ab titers were observed in the iFMDV–ISPA group. Due to a decreases in VNT, a second dose was administered to cattle, which resulted in an increase (*p* < 0.05) at 76 dpv in the seroneutralizing Abs titers in the iFMDV–ISPA group as compared to the iFMDV group. Remarkably, these VNT values were similar to the VNT induced by the commercial vaccine group. These values are associated with an 80% Expected Percentage os Protection (20). EPP calculates the probability that livestock would be protected against a 10 000 bovine infectious doses challenge after vaccination (1).

**Table 3:** Virus neutralizing antibody titers at 30, 48, and 76 days after inoculating cattle with different vaccines.

	**VNT (mean of Ab titers ± standard deviation)**		
**Vaccine**	**30 dpv**	**48 dpv**	**76 dpv**
iFMDV	1.02 ± 0.02	0.85 ± 0.03	1.4 ± 0.1
iFMDV–ISPA	1.8 ± 0.2^*^	1.2 ± 0.1^*^	2.2 ± 0.4^*^
Commercial	2.1 ± 0.2^**^	2.0 ± 0.4^**^	2.6 ± 0.2^**^
PBS	<1.0	<1.0	<1.0

Titers are expressed as log_10_ of the reciprocal of the serum dilution that neutralizes 50% of 100 TCID_50_ infective FMDV, using the fixed virus–variable serum method. Significant differences against iFMDV group:

* p < 0.05;

** *p < 0.01*.

### Immunization With iFMDV–ISPA Induces a Specific Cellular Immune Response Against FMDV in Cattle

**Figure 7:**
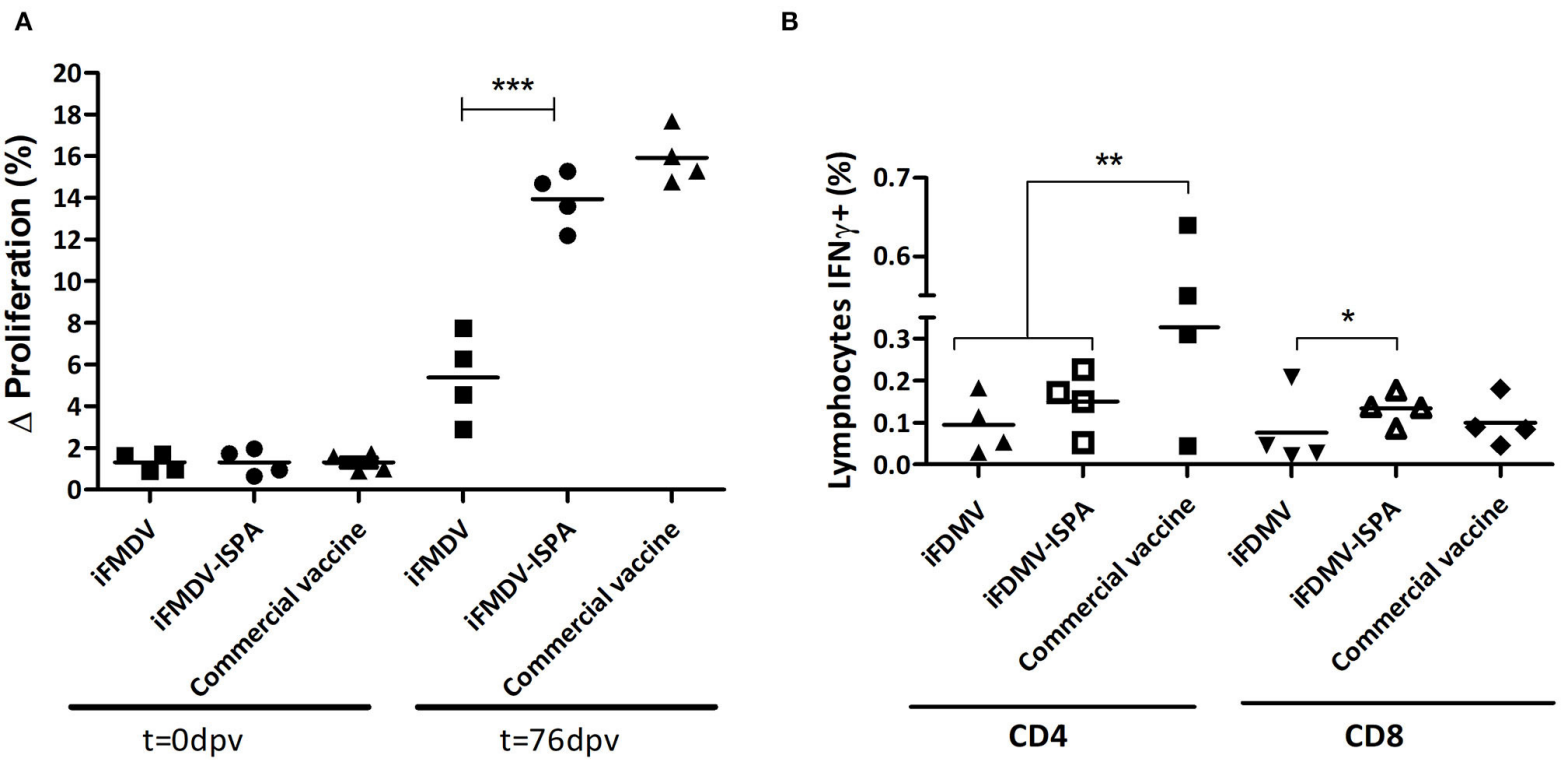
Cellular immune response in cattle. **(A)** Lymphocyte proliferative response after stimulation with iFMDV, A/Arg/2001 serotype, measured by CFSE loss. Differences (Δ%) were calculated as (% proliferating PBMCs stimulated with inactivated virus—% proliferating PBMCs without stimuli). **(B)** Percentages of CD8+/IFNγ^+^ or CD4+/IFNγ^+^ T cells in PBMCs of cattle immunized with iFMDV or iFMDV–ISPA, at 76 dpv. ***p < 0.001; **p < 0.01; *p < 0.05.

When PBMCs from vaccinated calves were stimulated with iFMDV, a significantly increased lymphoproliferative response (*p* < 0.001) was evident in iFMDV–ISPA compared to iFMDV (Figure 7A). No significant differences were detected between the iFMDV–ISPA and the commercial vaccine group (*p* = 0.075).

On the other hand, when lymphocytes stained with anti-bovine CD4, anti-bovine CD8, and anti-bovine INF-γ and then studied by flow cytometry, the percentages of IFNγ+/CD8+ lymphocytes from iFMDV–ISPA vaccinated calves were higher than in animals vaccinated with iFMDV alone (*p* < 0.05) (Figure 7B). Concerning CD4+ lymphocytes, a tendency of an increased production of IFNy was also observed in the iFMDV–ISPA group as compared to the iFMDV group, although the difference was not statistically significant (*p* = 0.72).

On the other hand, at 76 dpv, there were no statistically significant differences in the amounts of γδ T cells or IFN+/γδ T cells in the iFMDV–ISPA-immunized with respect to the iFMDV-immunized calves (data not shown).

## Discussion

In this work, we used a mouse model to examine the capacity of an iFMDV formulation containing new cage-like particles (ISPA), as a new generation adjuvant, to elicit a protective and specific immune response to FMDV. The results of the immunological immune response profile obtained in the murine model were confirmed in calves.

In the murine model, all animals vaccinated with iFMDV–ISPA were protected against homologous viral challenge while the protection percentages induced by a non-adjuvanted iFMDV vaccine were inferior. Individuals vaccinated with ISPA alone were not protected against viral challenge, showing that the protective response corresponded to an adaptive response against the virus and was not due to innate immune mechanisms induced by the adjuvant.

Total and seroneutralizing Abs against FMDV were significantly elevated in mice that received iFMDV–ISPA as compared to the group vaccinated with iFMDV alone. These results correlate with the protection induced on challenge. It is noteworthy that neutralizing antibody titers showed a good correlation with protection levels, substantiating the notion that they are an *in vitro* reflection of the immune response that occurs *in vivo* (1, 21, 22).

In addition, all isotypes of specific IgG were increased in iFMDV–ISPA group as compared to the group vaccinated with iFMDV alone, being IgG2a-b/IgG1 ratio also higher. It has been reported that murine macrophages could have a virus clarifying action by complement-fixing isotypes IgG2a, IgG2b, and IgG3 (5, 21, 23). The FcγI receptor (FcγRI), expressed in dendritic cells, monocytes and macrophages binds to these isotypes (24, 25). According to Klaus et al. (26) and Kipps et al. (27), IgG2a and IgG2b are the most effective isotypes in complement activation as well as in antibody-mediated cellular immune responses. Using the murine model to evaluate the quality of FMDV vaccines, Gnazzo et al. (6) reported that vaccine protection is associated not only with total FMDV antibody levels but also with the IgG2b/IgG1 ratio and the avidity of sera. Moreover, it has been reported that mice inoculated with iFMDV plus some adjuvants generate a complement-fixing IgG profile that correlates with protection on FMDV challenge (3, 28).

When the specific cellular response to the virus was studied, an increased lymphoproliferative response was evident in mice immunized with iFMDV–ISPA. These results suggest that the ISPA adjuvant improves the adaptive immune response against FMDV, reaching results similar to those obtained with the commercial vaccine. Ostrowski et al. (29) and Langellotti et al. (30) reported that vaccination of mice with inactivated FMDV induces T-cell responses and has been shown to increase CD8+ numbers in the spleen. Moreover, ISPA-iFMDV formulation triggers proliferation and IFNγ production in FMDV-specific CD4+ and CD8+ T lymphocytes (data not shown). It is well-described that IFN-γ is involved in the isotype switch of immunoglobulins, leading to an increase in the IgG2a and IgG2b types (31). This result is in agreement with the high levels of IgG2a and IgG2b obtained and the protection levels observed in the iFMDV–ISPA group. Previous work describes that ISCOMs improve the dendritic cross-presentation (9, 32–34). These data indicate that iFMDV adjuvanted with ISPA generates a strong cellular response, in accord with previous reports of studies that used cage-like particles.

Similar to what was observed in mice, the iFMDV–ISPA formulation generated an increase in anti-FMDV antibody titers in calves as compared to the iFMDV vaccine alone. In addition, animals immunized with iFMDV–ISPA displayed similar VNT titers as those immunized with a commercial vaccine approved by SENASA for vaccination in Argentina. Noteworthy, the commercial vaccine contains FMDV serotype A24/Cruzeiro, A/A2001, O1 Campos, and A/Arg2000, all of which bear epitopes that participate in the immune response against FMDV.

In cattle, numerous studies show a correlation between antibody titers against FMDV elicited by vaccination and *in vitro* and *in vivo* protection on experimental viral challenge. These correlations have allowed estimation of the Expected Percentage Protection to the homologous infection using titers of systemic α-FMDV Ab measured by liquid-phase ELISA or viral seroneutralization (19; 21; 1). Total and neutralizing anti-FMDV Ab titers reached in the iFMDV–ISPA group correspond to an EPP above 80% (35, 36). Importantly, an acceptable inactivated vaccine should induce 75% protection in cattle (1). Moreover, in cattle, IgG1 and IgG2 isotype titers were higher when ISPA was included as adjuvant in iFMDV vaccines. Bovine macrophages and neutrophils possess an immunoglobulin receptor to which IgG2 can bind (37). However, there are reports in which high IgG1 titers were related to high protection against FMDV challenge (38, 39). IgG1 is involved in both pathogen opsonization and seroneutralization in bovines. The particular role of each bovine IgG isotype in the response against FMDV has not been deeply characterized yet. In addition, IgG1/IgG2 ratio > 1 is related to FMDV protection and it is used as a protection parameter when there are low VNTs (39, 40).

Regarding cellular responses, *in vitro* T-cell stimulation was significantly higher in cattle PBMCs of the iFMDV–ISPA group than of the iFMDV group. In addition, IFNγ production was increased in CD8+ PBMCs derived from iFMDV–ISPA-immunized cattle. Thus, we here demonstrate that the ISPA-FMDV vaccine induces a cellular immune response in these bovines by inducing IFNγ secretion and raising viral-specific PBMC proliferation. Moreover, IgG1 is usually taken as a parameter of cellular immune response activation (41, 42).

The role of FMDV cellular immunity responses in a target species, such as the bovine, is still unclear, although many reports indicate its relevance to fight the infection. In this way, specific T-cell-mediated antiviral responses have been observed in cattle after infection or vaccination (43–45). Also, FMDV vaccination induces rapid T-cell responses, and FMDV-specific CD4+ T-cell proliferation has been detected as early as 7 dpv (46). T-helper cells are necessary for the induction of isotype switching to generate high-affinity antibodies and to reach a protective neutralizing response to vaccination with iFMDV (47). On the other hand, CD8+ T-cell–mediated immune responses to FMDV have been reported in pigs (45, 48, 49) and cattle (43, 50, 51). Vaccination with the conventional iFMDV vaccine induces circulating memory CD8+ T cells which, upon an appropriate stimulus, can be expanded and are cytotoxic (51). Stenfeld et al. (52) demonstrated the role of a CTL response in preventing the FMDV carrier state in vaccinated cattle. Besides, the percentage of CD4+ lymphocytes and the CD4/CD8 ratio after vaccination may serve as a parameter to select young sires with a high immune response against FMDV (53). Moreover, IFN-γ displays activity against FMDV (54), by controlling viral replication and spreading within the host through natural killer cell and macrophage activation (55). Thus, a positive correlation between IFN-γ response and vaccine-induced protection as well as reduction of long-term persistence of FMDV has been observed in cattle (56).

Cattles numbers included in this pilot study was equal to those used in other preliminary studies on vaccine candidates (48, 57–59), although it is not enough for statistical analysis (60). However, the results obtained serve as a proof of concept of the usefulness of ISPA as adjuvant for FMDV vaccines.

Future work will be devoted to examining whether vaccine formulations containing ISPA promote the virus presentation to the immune effectors, and in this way enhance the immune response generated and the protection obtained. Some authors have reported that ISCOMs induce local recruitment, activation, and maturation of immune cells, such as dendritic cells; granulocytes; F4/80 int cells; and T, B, and NK cells (10, 61, 62), increasing in this way the chances of the antigen to come into contact with immune cells. In addition, Brok et al. (34) proved that saponin-based adjuvants enhance antigen cross-presentation by dendritic cells and T-cell activation. Moreover, Prochetto et al. have proved that a vaccine for *Trypanosoma cruzi* formulated with ISPA and a recombinant trans-sialidase fraction favorably modulates the regulatory arm of the immune system to reach immune protection against the parasite (13).

In conclusion, ISPA displays an important adjuvant activity for FMDV vaccines, increasing and modulating the humoral and cellular responses in vaccinated mice and cattle and yielding enhanced protection against challenge.

## Data Availability Statement

The data are available on request to the corresponding author.

## Ethics Statement

The animal study was reviewed and approved by Comité Institucional para el Cuidado y Uso de Animales de Experimentación (CICUAE) - Centro de Investigación en Ciencias Veterinarias y Agronómicas del INTA.

## Author Contributions

JB: collaboration in work designing, acquisition, analysis, interpretation of field and laboratory data, drafting, final approval of the version to be published, and ensuring that questions related to the accuracy or integrity of the work were appropriately investigated and resolved. CK, MG, VG, IS, CL, CM, RG, LC, GL, VQ, and IM: acquisition and analysis of laboratory data for the work, critical revision for intellectual content, final approval of the version to be published, ensuring that questions related to the accuracy, and integrity of laboratory work were appropriately investigated and resolved. PZ: conception and design of the work, critical revision of the work for important intellectual content, final approval of the version to be published, ensuring that questions related to the accuracy, and integrity of the work were appropriately investigated and resolved. All authors: contributed to the article and approved the submitted version.

## Conflict of Interest

The authors declare that the research was conducted in the absence of any commercial or financial relationships that could be construed as a potential conflict of interest.

## Supplementary Material

Supplementary Figure 1 The figure shows representative dot plots used for selecting the lymphocyte region based on side sideward scatter (SSC) on the *y*-axis and forward side scatter (FSC) on the *x*-axis. Lymphocyte proliferative response after stimulation with iFMDV is shown. **(A)** Representative dot plots from mice splenocytes at 0 dpv and CFSE loss (H-3 gate). **(B)** Representative dot plots from mice splenocytes at 21 dpv and CFSE loss (H-3 gate). **(C)** Representative dot plots from bovine PBMCs at 0 dpv and CFSE loss (H-3 gate). **(D)** Representative dot plots from bovine PBMCs at 76 dpv and CFSE loss (H-3 gate).

Supplementary Figure 2 The figure shows representative dot plots, from bovine PBMCs, used for selecting the lymphocyte region based on side sideward scatter (SSC) on the *y*-axis and forward side scatter (FSC) on the *x*-axis. Then, we selected the CD8 region based on fluorescence anti-CD8 stain on the *y*-axis and CD4 region based on fluorescence anti-CD4 stain on the *x*-axis. PBMCs incubated for 18 h with iFMDV are shown.

## References

[1] OIE (World Organisation for Animal Health) Foot and Mouth Disease (infection with FMDV). In OIE editor. OIE Terrestrial Manual. Paris: OIE (2012). p. 1–32. Available at: https://books.google.com.ar/books?id=l5b5AAAACAAJ

[2] Mattion N Konig G Seki C Smitsaart E Maradei E Robiolo B. Reintroduction of foot-and-mouth disease in Argentina: characterisation of the isolates and development of tools for the control and eradication of the disease. Vaccine. (2004) 22:4149–62. 10.1016/j.vaccine.2004.06.040 15474705

[3] Batista A Quattrocchi V Olivera V Langellotti C Pappalardo JS Di Giacomo S. Adjuvant effect of Cliptox^TM^ on the protective immune response induced by an inactivated vaccine against foot and mouth disease virus in mice. Vaccine. (2010) 28:6361–6. 10.1016/j.vaccine.2010.06.098 20637310

[4] Zamorano P Decheneux C Quattrocchi V Olivera V Langellotti C DiGiacomo S. Vaccination against Foot-and-Mouth Disease, association between humoral immune response in cattle and mice. In: OIE-IABS editor. Practical Alternatives to Reduce Animal Testing in Quality Control of Veterinary Biologicals in the Americas. Buenos Aires: OIE-IABS (2010). p. 100–11.

[5] Quattrocchi V Langellotti C Pappalardo JS Olivera V Di Giacomo S van Rooijen N. Role of macrophages in early protective immune responses induced by two vaccines against foot and mouth disease. Antiviral Res. (2011) 92:262–70. 10.1016/j.antiviral.2011.08.007 21878353

[6] Gnazzo V Quattrocchi V Soria I Pereyra E Langellotti C Pedemonte A. Mouse model as an efficacy test for foot-and-mouth disease vaccines. Transbound Emerg Dis. (2020) [Epub ahead of print]. 10.1111/tbed.13591 32320534

[7] Mohan T Verma P Rao DN. Novel adjuvants & delivery vehicles for vaccines development: a road ahead. Indian J Med Res. (2013). 138:779–95. Available at: http://www.ncbi.nlm.nih.gov/pubmed/24434331 24434331 PMC3928709

[8] Singh M (ed.) (2007). Vaccine Adjuvants and Delivery Systems. Emeryville, CA: John Wiley & Sons, Inc Available online at: https://books.google.com.ar/books?id=7QKRrTPwuDYC 10.1002/9780470134931

[9] Maraskovsky E Schnurr M Wilson NS Robson NC Boyle J Drane D. Development of prophylactic and therapeutic vaccines using the ISCOMATRIX adjuvant. Immunol Cell Biol. (2009) 87:371–6. 10.1038/icb.2009.21 19381160

[10] Sun H-X Xie Y Ye Y-P. ISCOMs and ISCOMATRIX. Vaccine. (2009) 27:4388–401. 10.1016/j.vaccine.2009.05.032 19450632

[11] Bertona D Pujato N Bontempi I Gonzalez V Cabrera G Gugliotta L. Development and assessment of a new cage-like particle adjuvant. J Pharm Pharmacol. (2017) 69:1293–303. 10.1111/jphp.12768 28664569

[12] Morein B Sundquist B Höglund S Dalsgaard K Osterhaus A. Iscom, a novel structure for antigenic presentation of membrane proteins from enveloped viruses. Nature. (1984) 308:457–60. 10.1038/308457a0 6709052

[13] Prochetto E Roldán C Bontempi IA Bertona D Peverengo L Vicco MH Trans-sialidase-based vaccine candidate protects against Trypanosoma cruzi infection, not only inducing an effector immune response but also affecting cells with regulatory/suppressor phenotype. Oncotarget. (2017) 8:58003–20. 10.18632/oncotarget.18217 28938533 PMC5601629

[14] Fernández FM Borca MV Sadir AM Fondevila N Mayo J Schudel AA. Foot-and-mouth disease virus (FMDV) experimental infection: susceptibility and immune response of adult mice. Vet Microbiol. (1986) 12:15–24. 10.1016/0378-1135(86)90037-4 3014713

[15] D'Antuono A Laimbacher AS La Torre J Tribulatti V Romanutti C Zamorano P. HSV-1 amplicon vectors that direct the *in situ* production of foot-and-mouth disease virus antigens in mammalian cells can be used for genetic immunization. Vaccine. (2010) 28:7363–72. 10.1016/j.vaccine.2010.09.011 20851082

[16] Quattrocchi V Pappalardo JS Langellotti C Smitsaart E Fondevila N Zamorano P. Early protection against foot-and-mouth disease virus in cattle using an inactivated vaccine formulated with Montanide ESSAI IMS D 12802 VG PR adjuvant. Vaccine. (2014) 32:2167–72. 10.1016/j.vaccine.2014.02.061 24631088

[17] Hamblin C Barnett ITR Hedger RS. A new enzyme-linked immunosorbent assay (ELISA) for the detection of antibodies against foot-and-mouth disease virus I. Development and method of ELISA. J Immunol Methods. (1986). 93:115–21. 10.1016/0022-1759(86)90441-2 3021854

[18] Romera SA Puntel M Quattrocchi V Zajac PDM Zamorano P Blanco Viera J Protection induced by a glycoprotein E-deleted bovine herpesvirus type 1 marker strain used either as an inactivated or live attenuated vaccine in cattle. BMC Vet Res. (2014) 10:8 10.1186/1746-6148-10-8 24401205 PMC3896737

[19] Quattrocchi V Bianco V Fondevila N Pappalardo S Sadir A Zamorano P. Use of new adjuvants in an emergency vaccine against foot-and-mouth disease virus: evaluation of conferred immunity. Dev. Biol. (Basel). (2004). 119:481–97. Available at: http://www.ncbi.nlm.nih.gov/pubmed/15742663.s 15742663

[20] Maradei E La Torre J Robiolo B Esteves J Seki C Pedemonte A. Updating of the correlation between lpELISA titers and protection from virus challenge for the assessment of the potency of polyvalent aphtovirus vaccines in Argentina. Vaccine. (2008) 26:6577–86. 10.1016/j.vaccine.2008.09.033 18835312

[21] McCullough KC Bruckner L Schaffner R Fraefel W Muller HK Kihm U. Relationship between the anti-FMD virus antibody reaction as measured by different assays, and protection *in vivo* against challenge infection. Vet Microbiol. (1992) 30:99–112. 10.1016/0378-1135(92)90106-4 1313624

[22] Mattion N Goris N Willems T Robiolo B Maradei E Beascoechea CP. Some guidelines for determining foot-and-mouth disease vaccine strain matching by serology. Vaccine. (2009) 27:741–7. 10.1016/j.vaccine.2008.11.026 19041355

[23] Rigden RC Carrasco CP Summerfield A McCullough KC. Macrophage phagocytosis of foot-and-mouth disease virus may create infectious carriers. Immunology. (2002) 106:537–48. 10.1046/j.1365-2567.2002.01460.x 12153517 PMC1782748

[24] van der Poel WH Hage JJ. Spread of an intramuscularly administered live gE-negative BHV1 marker vaccine in 2 cattle farms. Tijdschr Diergeneeskd. (1998) 123:109–11. 9537086

[25] Habiela M Seago J Perez-Martin E Waters R Windsor M Salguero FJ. Laboratory animal models to study foot-and-mouth disease: a review with emphasis on natural and vaccine-induced immunity. J Gen Virol. (2014) 95:2329–45. 10.1099/vir.0.068270-0 25000962 PMC4202264

[26] Klaus GG Pepys MB Kitajima K Askonas BA. Activation of mouse complement by different classes of mouse antibody. Immunology. (1979). 38:687–95. Available at: https://www.ncbi.nlm.nih.gov/pubmed/521057. 521057 PMC1457877

[27] Kipps TJ Parham P Punt J Herzenberg LA. Importance of immunoglobulin isotype in human antibody-dependent, cell-mediated cytotoxicity directed by murine monoclonal antibodies. J. Exp. Med. (1985). 161:1–17. 10.1084/jem.161.1.1 3918141 PMC2187540

[28] Pérez Filgueira DM Berinstein A Smitsaart E Borca MV Sadir AM Isotype profiles induced in Balb/c mice during foot and mouth disease (FMD) virus infection or immunization with different FMD vaccine formulations. Vaccine. (1995) 13:953–60. 10.1016/0264-410X(95)00078-F 7483770

[29] Ostrowski M Vermeulen M Zabal O Geffner JR Sadir AM Lopez OJ. Impairment of thymus-dependent responses by murine dendritic cells infected with foot-and-mouth disease virus. J Immunol. (2005) 175:3971–9. 10.4049/jimmunol.175.6.3971 16148145

[30] Langellotti C Quattrocchi V Alvarez C Ostrowski M Gnazzo V Zamorano P. Foot-and-mouth disease virus causes a decrease in spleen dendritic cells and the early release of IFN-α in the plasma of mice. Differences between infectious and inactivated virus. Antiviral Res. (2012) 94:62–71. 10.1016/j.antiviral.2012.02.009 22387627

[31] Abbas AK Lichtman AH Pillai S Cellular and Molecular Immunology. 7th ed Philadelphia, PA: Elsevier/Saunders (2012).

[32] Wilson NS Yang B Morelli AB Koernig S Yang A Loeser S. ISCOMATRIX vaccines mediate CD8 T-cell cross-priming by a MyD88-dependent signaling pathway. Immunol Cell Biol. (2012) 90:540–52. 10.1038/icb.2011.71 21894173 PMC3365289

[33] Wilson NS Duewell P Yang B Li Y Marsters S Koernig S. Inflammasome-615 dependent and -independent IL-18 production mediates immunity to the ISCOMATRIX 616 adjuvant. J. Immunol. (2014). 192:3259–68. 10.4049/jimmunol.1302011 24610009

[34] Den Brok MH Büll C Wassink M De Graaf AM Wagenaars JA Minderman M. Saponin-based adjuvants induce cross-presentation in dendritic cells by intracellular lipid body formation. Nat Commun. (2016) 7:13324. 10.1038/ncomms13324 27819292 PMC5103066

[35] Robiolo B La Torre J Maradei E Beascoechea CP Perez A Seki C. Confidence in indirect assessment of foot-and-mouth disease vaccine potency and vaccine matching carried out by liquid phase ELISA and virus neutralization tests. Vaccine. (2010) 28:6235–41. 10.1016/j.vaccine.2010.07.012 20643090

[36] Senasa Servicio Nacional de Sanidad y Calidad Agroalimentaria Res 609/2017. CABA (2017).

[37] Tizard I Inmunologí a Veterinaria. 5th ed Mexico: McGraw-Hill; Interamericana (1998).

[38] Mulcahy G Gale C Robertson P Iyisan S DiMarchi RD Doel TR. Isotype responses of infected, virus-vaccinated and peptide-vaccinated cattle to foot-and-mouth disease virus. Vaccine. (1990) 8:249–56. 10.1016/0264-410X(90)90054-P 2163575

[39] Capozzo AVE Periolo OH Robiolo B Seki C La Torre JL Grigera PR Total and isotype humoral responses in cattle vaccinated with foot and mouth disease virus (FMDV) immunogen produced either in bovine tongue tissue or in BHK-21 cell suspension cultures. Vaccine. (1997) 15:624–30. 10.1016/S0264-410X(96)00284-8 9178462

[40] Lavoria MA Di-Giacomo S Bucafusco D Franco-Mahecha OL Pérez-Filgueira DM Capozzo AV. Avidity and subtyping of specific antibodies applied to the indirect assessment of heterologous protection against Foot-and-Mouth Disease Virus in cattle. Vaccine. (2012) 30:6845–50. 10.1016/j.vaccine.2012.09.011 23000129

[41] Clerici M Shearer GM. The Th1-Th2 hypothesis of HIV infection: new insights. Immunol Today. (1994) 15:575–81. 10.1016/0167-5699(94)90220-8 7848519

[42] Sin JI Kim JJ Weiner DB Arnold RL Shroff KE McCallus D. IL-12 gene as a DNA vaccine adjuvant in a herpes mouse model: IL-12 enhances Th1-type CD4+ T cell-mediated protective immunity against herpes simplex virus-2 challenge. J Immunol. (1999) 162:2912–21. 10072541

[43] Childerstone AJ Cedillo-Baron L Foster-Cuevas M Parkhouse RME. Demonstration of bovine CD8+ T cell responses to foot-and-mouth disease virus. J Gen Virol. (1999) 80:663–9. 10.1099/0022-1317-80-3-663 10092006

[44] Bautista EM Ferman GS Golde WT. Induction of lymphopenia and inhibition of T cell function during acute infection of swine with foot and mouth disease virus (FMDV). Vet Immunol Immunopathol. (2003). 10.1016/S0165-2427(03)00004-7 12628764

[45] Patch JR Kenney M Pacheco JM Grubman MJ Golde WT. Characterization of cytotoxic T lymphocyte function after foot-and-mouth disease virus infection and vaccination. Viral Immunol. (2013). 26:239–49. 10.1089/vim.2013.0011 23829779 PMC3739958

[46] Doel TR Williams L Barnett PV. Emergency vaccination against foot-and-mouth disease: Rate of development of immunity and its implications for the carrier state. Vaccine. (1994). 10.1016/0264-410X(94)90262-3 8085375

[47] Carr BV Lefevre EA Windsor MA Inghese C Gubbins S Prentice H. CD4+ T-cell responses to foot-and-mouth disease virus in vaccinated cattle. J Gen Virol. (2013) 94:97–107. 10.1099/vir.0.045732-0 23034593 PMC3542717

[48] Blanco E Garcia-Briones M Sanz-Parra A Gomes P De Oliveira E Valero ML. Identification of T-cell epitopes in nonstructural proteins of foot-and-mouth disease virus. J Virol. (2001) 75:3164–74. 10.1128/JVI.75.7.3164-3174.2001 11238843 PMC114110

[49] García-Briones MM Blanco E Chiva C Andreu D Ley V Sobrino F. Immunogenicity and T cell recognition in swine of foot-and-mouth disease virus polymerase 3D. Virology. (2004). 10.1016/j.virol.2004.01.027 15110524

[50] Guzman E Taylor G Charleston B Skinner MA Ellis SA. An MHC-restricted CD8+ T-cell response is induced in cattle by foot-and-mouth disease virus (FMDV) infection and also following vaccination with inactivated FMDV. J Gen Virol. (2008). 89:667–75. 10.1099/vir.0.83417-0. 18272757

[51] Guzman E Taylor G Charleston B Ellis SA. Induction of a cross-reactive CD8+ T cell response following foot-and-mouth disease virus vaccination. J Virol. (2010) 84:12375–84. 10.1128/JVI.01545-10 20861264 PMC2976409

[52] Stenfeldt C Eschbaumer M Smoliga GR Rodriguez LL Zhu J Arzt J. Clearance of a persistent picornavirus infection is associated with enhanced pro-apoptotic and cellular immune responses. Sci. Rep. (2017). 7:1–15. 10.1038/s41598-017-18112-4 29259271 PMC5736604

[53] Li X Meng X Wang S Li Z Yang L Tu L. Virus-like particles of recombinant PCV2b carrying FMDV-VP1 epitopes induce both anti-PCV and anti-FMDV antibody responses. Appl Microbiol Biotechnol. (2018) 102:10541–50. 10.1007/s00253-018-9361-2 30338355

[54] Summerfield A Guzylack-Piriou L Harwood L McCullough KC. Innate immune responses against foot-and-mouth disease virus: current understanding and future directions. Vet Immunol Immunopathol. (2009) 15:205–10. 10.1016/j.vetimm.2008.10.296 19026453

[55] Zhang ZD Hutching G Kitching P Alexandersen S. The effects of gamma interferon on replication of foot-and-mouth disease virus in persistently infected bovine cells. Arch Virol. (2002) 147:2157–67. 10.1007/s00705-002-0867-6 12417950

[56] Oh Y Fleming L Statham B Hamblin P Barnett P Paton DJ. Interferon-γ induced by *in vitro* re-stimulation of CD4+ T-cells correlates with *in vivo* FMD vaccine induced protection of cattle against disease and persistent infection. PLoS ONE. (2012) 7:e44365. 10.1371/journal.pone.0044365 23028529 PMC3460943

[57] Bittle JL Houghten RA Alexander H Shinnick TM Sutcliffe JG Lerner RA. Protection against foot-and-mouth disease by immunization with a chemically synthesized peptide predicted from the viral nucleotide sequence. Nature. (1982) 298:30–3. 10.1038/298030a0 7045684

[58] Bachmann MF Zinkernagel RM. Neutralizing antiviral B cell responses. Annu Rev Immunol. (1997) 15:235–70. 10.1146/annurev.immunol.15.1.235 9143688

[59] Lee BO Rangel-Moreno J Moyron-Quiroz JE Hartson L Makris M Sprague F. CD4 T cell-independent antibody response promotes resolution of primary influenza infection and helps to prevent reinfection. J Immunol. (2005) 175:5827–38. 10.4049/jimmunol.175.9.5827 16237075

[60] Soria I Quattrocchi V Langellotti C Gammella M Digiacomo S Garcia de la Torre B. Dendrimeric peptides can confer protection against foot-and-mouth disease virus in cattle. PLoS ONE. (2017) 12:e0185184. 10.1371/journal.pone.0185184 28949998 PMC5614567

[61] Reed SG Bertholet S Coler RN Friede M. New horizons in adjuvants for vaccine development. Trends Immunol. (2009) 30:23–32. 10.1016/j.it.2008.09.006 19059004

[62] Reimer JM Karlsson KH Lövgren-Bengtsson K Magnusson SE Fuentes A Stertman L. Matrix-m™ adjuvant induces local recruitment, activation and maturation of central immune cells in absence of antigen. PLoS ONE. (2012) 7:e41451. 10.1371/journal.pone.0041451 22844480 PMC3402407

