# Supplementary figures and images for: A New Cage-Like Particle Adjuvant Enhances Protection of Foot-and-Mouth Disease Vaccine
Source: Front Vet Sci. 2020 Jul 31;7:396. doi: 10.3389/fvets.2020.00396 (PMC7411152; doi:10.3389/fvets.2020.00396)

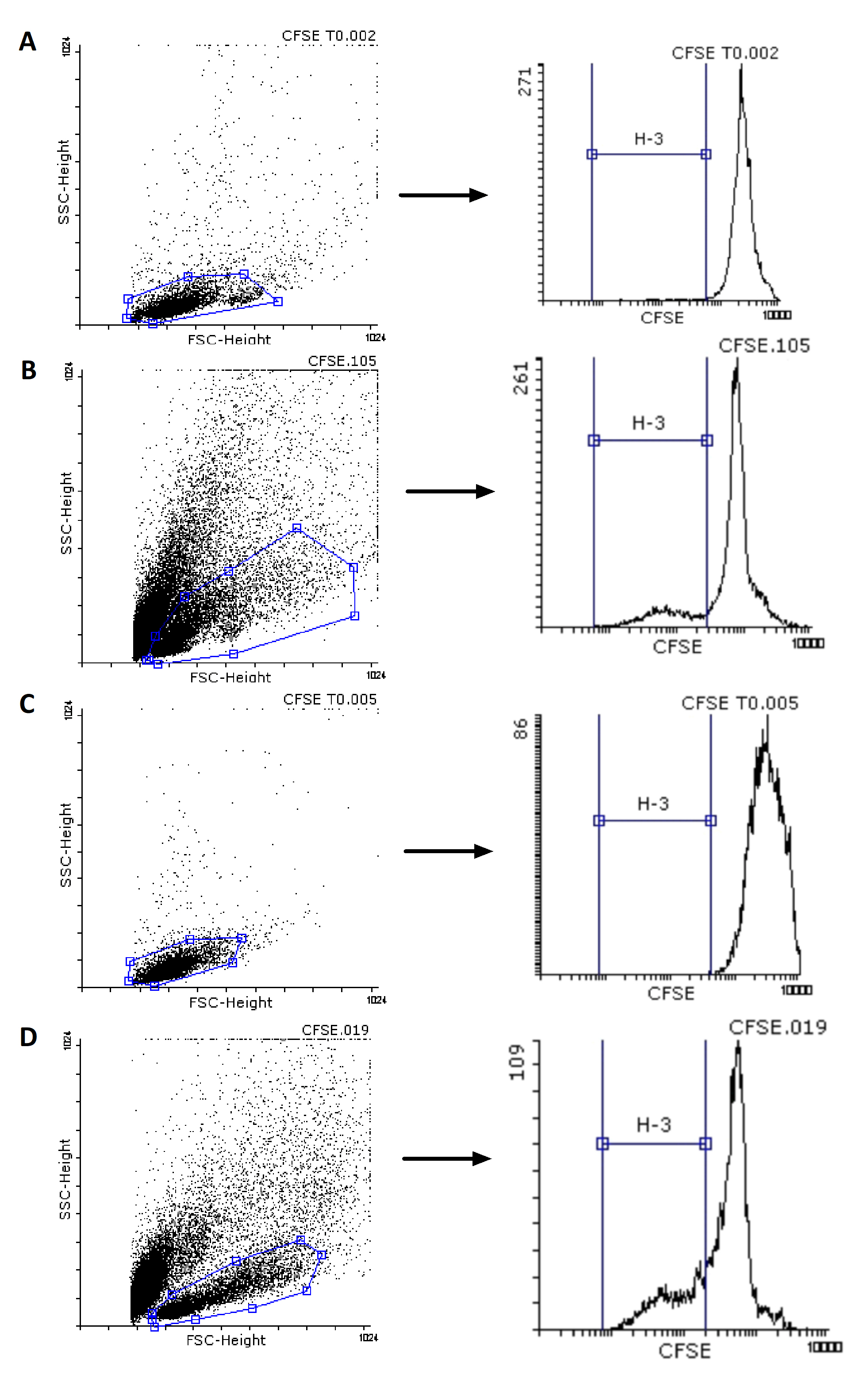

Supplement: Supplementary Figure 1 — The figure shows representative dot plots used for selecting the lymphocyte region based on side sideward scatter (SSC) on the y-axis and forward side scatter (FSC) on the x-axis. Lymphocyte proliferative response after stimulation with iFMDV is shown. (A) Representative dot plots from mice splenocytes at 0 dpv and CFSE loss (H-3 gate). (B) Representative dot plots from mice splenocytes at 21 dpv and CFSE loss (H-3 gate). (C) Representative dot plots from bovine PBMCs at 0 dpv and CFSE loss (H-3 gate). (D) Representative dot plots from bovine PBMCs at 76 dpv and CFSE loss (H-3 gate). [file Image_1.JPEG]

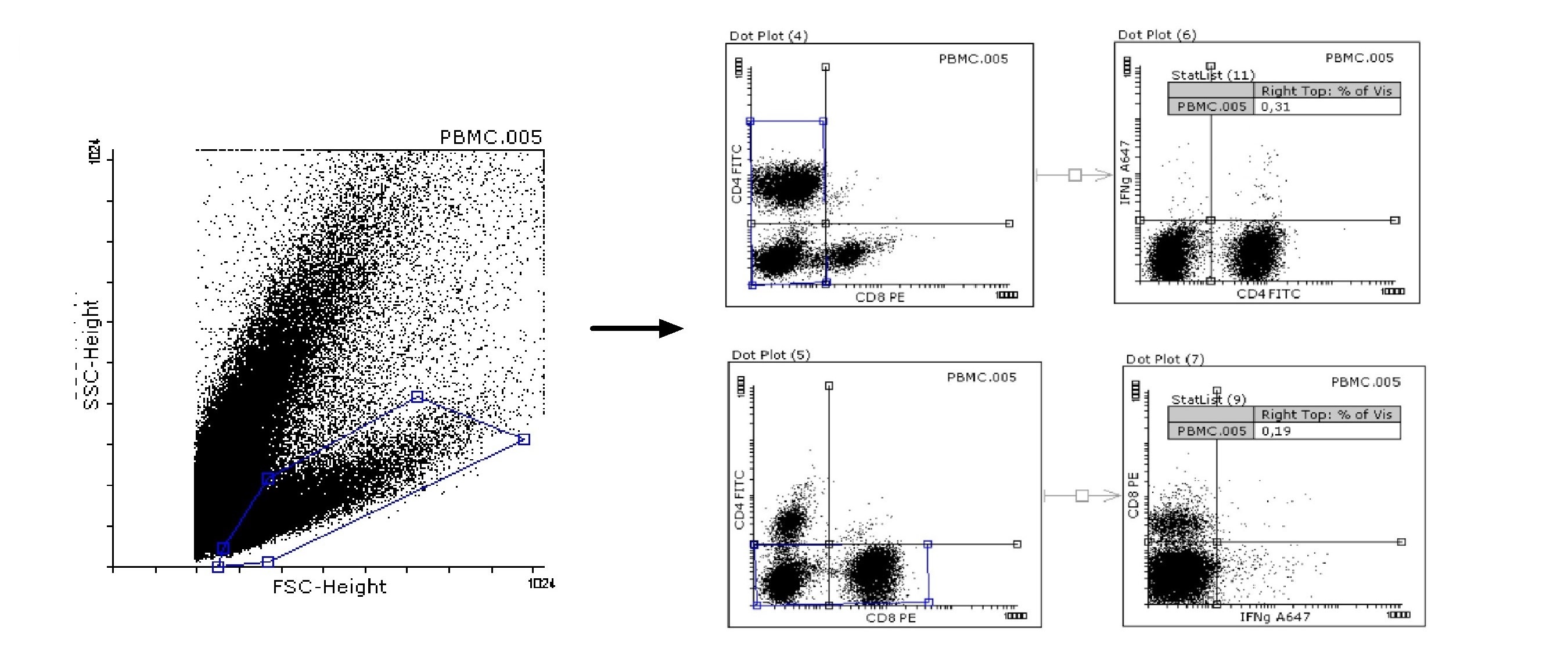

Supplement: Supplementary Figure 2 — The figure shows representative dot plots, from bovine PBMCs, used for selecting the lymphocyte region based on side sideward scatter (SSC) on the y-axis and forward side scatter (FSC) on the x-axis. Then, we selected the CD8 region based on fluorescence anti-CD8 stain on the y-axis and CD4 region based on fluorescence anti-CD4 stain on the x-axis. PBMCs incubated for 18 h with iFMDV are shown. [file Image_2.JPEG]
